# Can quinoa (*Chenopodium quinoa*) replace traditional cereals under current climate scenarios?

**DOI:** 10.3389/fpls.2025.1636565

**Published:** 2025-08-21

**Authors:** Hongju Sun, Waqas ud Din Khan, Mohsin Tanveer, Usman Ijaz, Zhanyuan Lu, Sergey Shabala

**Affiliations:** ^1^ School of Life Sciences, Inner Mongolia University, Hohhot, Inner Mongolia, China; ^2^ Key Laboratory of Forage and Endemic Crop Biotechnology, Ministry of Education, Hohhot, Inner Mongolia, China; ^3^ Xinjiang Institute of Ecology and Geography, Chinese Academy of Sciences, Urumqi, China; ^4^ School of Biological Sciences, The University of Western Australia, Perth, Australia; ^5^ Department of Agriculture, Government College University, Lahore, Pakistan; ^6^ Tasmanian Institute of Agriculture, University of Tasmania, Launceston, TAS, Australia; ^7^ Inner Mongolia Academy of Agricultural and Animal Husbandry Sciences, Hohhot, China; ^8^ Inner Mongolia Key Laboratory of Degradation Farmland Ecological Remediation and Pollution Control, Hohhot, China; ^9^ International Research Centre for Environmental Membrane Biology, Foshan University, Foshan, China

**Keywords:** salinity, drought, food security, climate change, abiotic stress, adaptation

## Abstract

Agriculture is extremely vulnerable to climate change and crop production is severely hampered by climate extremes. Not only does it cost growers over US$170Bln in lost production, but it also has major implications for global food security. In this study, we argue that, under current climate scenarios, agriculture in the 21^st^ century will become saline, severely limiting (or even making impossible) the use of traditional cereal crops for human caloric intake. As regaining the lost abiotic stress tolerance can only be achieved using modern gene editing technologies and given uncertainties on when and to what extent the public will embrace such new technologies, *de novo* domestication of already tolerant wild species or semi-domesticated “orphan” species is arguably the most efficient way to proceed. One of them is quinoa (*Chenopodium quinoa*), which is the focus of this review. Accordingly, we comprehensively evaluated the nutritional qualities of quinoa and discussed the benefits of using quinoa as a viable alternative to traditional cereals from both agronomical and nutritional points of view. We also highlight the existing gaps in the knowledge and the next steps required to ensure public acceptance of quinoa in a daily diet, alongside (or instead of) traditional cereals such as wheat or rice.

## 1 Cereal grain production under future climate scenarios

Agriculture is highly vulnerable to climate change, and crop production is severely hampered by climate extremes such as heat, drought, waterlogging, and salinity (Suzuki et al., 2014; Zandalinas and Mittler, 2022). Regardless of the nature of current global warming (e.g., anthropogenic vs. natural), the implications for the profitability and sustainability of agricultural production systems are substantial (Palmgren and Shabala, 2024) and already come at a massive cost. It was estimated that the overall loss in crop production from climate-driven abiotic stresses exceeds US$ 170 Bln p.a. and represents a major threat to global food security (Razzaq et al., 2021). Abiotic stress tolerance was present in wild relatives of modern crops but was lost (or very significantly weakened) during domestication (Doebley et al., 2006; Palmgren et al., 2015; Menguer et al., 2017; Yolcu et al., 2020; Rawat et al., 2022). In addition, both the intensity and frequency of extreme weather events will increase (Ebi et al., 2021; Barriopedro et al., 2023; González-Trujillo et al., 2023; Chen et al., 2024). Combined with the current trends in population growth and the extent of urbanization (at the expense of agricultural land), this poses a significant threat to global food security in the next decade(s).

The major primary climate-driven constraints on crop production are global warming and the associated increase in the severity and frequency of drought events. Currently, 40% of the entire land area of our planet is classified as drylands (Earth.org, 2024) and is increasing at an alarming rate (Vicente-Serrano et al., 2024). It is expected that by the end of this century, over 50% of the agricultural land will become arid or semi-arid and could be made produced only by irrigation. Right now, 70% of all cultivated land in Pakistan (the 5^th^ most populous country in the world) and 42% of land in India (the world’s most populous country) are on irrigation (Liu et al., 2020a), and this proportion is only going to increase. Putting it in plain language: in a decade, most of the world’s agriculture will become irrigated.

The increasing reliance on irrigation comes with the caveat of soil salinization. Good-quality irrigation water is a scarce resource. As a result, a significant amount of salt (between 3 and 6 tons; Liu et al., 2020a) is added to each hectare of irrigated land every year. At the same time, all major staple crops that provide a bulk of calories for humans (such as wheat, rice, maize, and soybeans) are highly salt-sensitive (see below). Being selected for reduced Na^+^ accumulation in the shoot, elite cultivars of these species exclude 95 to 97% of salt from uptake (Munns, 2002) causing a massive salinity build-up in the rhizosphere (see Liu et al., 2020a for modelling) and further exacerbating salinity issues. Therefore, mankind can rely on traditional cereals to meet their dietary needs under future climate scenarios.

**Figure 1:**
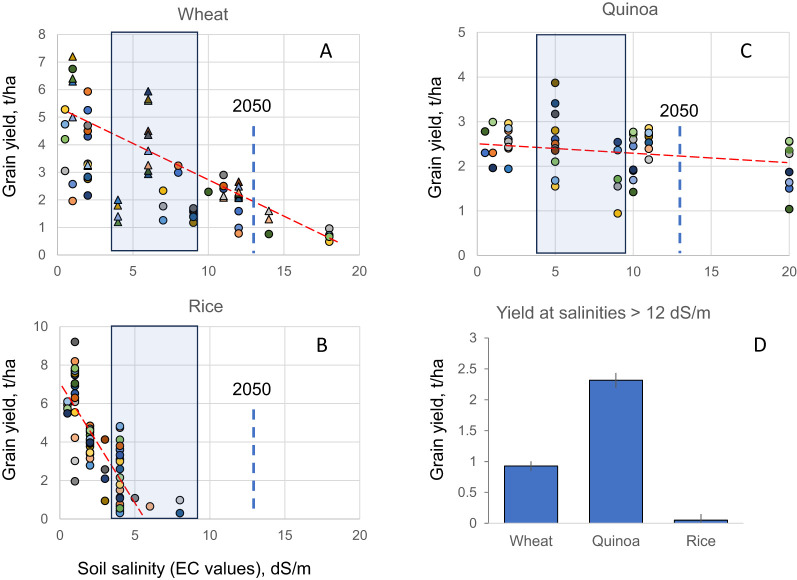
Effect of soil salinity on grain yield in wheat **(A)**, rice **(B)** and quinoa **(C)** species. Data is collated based on 11 papers describing performance of 38 genotypes for wheat; 9 papers/31 genotypes for rice; and 12 papers/35 genotypes for quinoa. Each symbol represents a specific genotype. In **(A)** circles represent bread wheat (*Triticum aestivum*) while triangles define durum (*Triticum turgidum* ssp. *durum*) wheat genotypes. The slopes of the red dotted lines illustrate differences in sensitivity of species to increasing soil salinity. Shaded boxes represent the current range of salinities experience by crops in major grain producing countries experiencing salinity issues (China, India, USA, Mexico, Pakistan, Australia). The bold vertical dotted line shows a predicted values of soil salinity in 2050 derived from trends in changes in EC values in irrigation water (based on Liu et al., 2020a). **(D)** - grain yield of three species grown at salinities predicted for 2050.

To answer this question, we conducted a meta-analysis of the literature summarizing effects of salinity on production of wheat and rice, two major cereal crops collectively provide ~ 40% of total calorie intake by humans. In both species, the grain yield showed a progressive dose-dependent decline with increasing salinity (**Figure 1**). Rice is extremely sensitive to salinity, with no grain yield produced at salinity levels exceeding 10 dS/m (an equivalent of 100 mM NaCl, or approximately 20% of seawater salt concentration; **Figure 1B**). Wheat is doing slightly better (**Figure 1A**); however, a 50% decline in grain yield is observed at salinities of around 9 dS/m, a level of salt found in irrigation water in most populous countries (e.g. China, India, USA, Mexico, Pakistan) affected by salinity (Liu et al., 2020a). Extrapolating the current trends in electrical conductivity of saline water (Liu et al., 2020a), one can predict that by 2050, the average salinity level in the soil will be within the 12–13 dS/m range (blue dotted line in **Figure 1** panels). These salinity levels will be not only incompatible with rice production, but also reduce wheat production by ~ 70% (**Figure 1A**). Thus, based on the weighted average contribution, the mankind will be short of ~39% calories produced by these two cereal crops.

## 2 What are alternatives?

Broadly, three possible options are available. One is the extensification of agriculture; that is, using more land for agricultural purposes. This option is not viable because most of the arable hands have *already* been used. The second option is to regain abiotic stress (and, specifically, salinity) tolerance lost during domestication (Rawat et al., 2022; Palmgren and Shabala, 2024). Termed as “rewilding” (Palmgren et al., 2015), this concept implies identification of genes important for survival in challenging environments (that were lost during the domestication process) in wild relatives and reintroduced into modern crops (Palmgren et al., 2015). This option is technically viable but will require broad public acceptance of products created using gene editing technologies, as well as legislative hurdles. Worldwide, consumers have limited understanding, misconceptions, and unfamiliarity with plant gene technologies in agriculture (Wunderlich and Gatto, 2015; Woźniak-Gientka et al., 2022) and the extent to which the general public will embrace such new technologies. In this context, *de novo* domestication of already-tolerant wild species or semi-domesticated “orphan” species is arguably the most efficient way to proceed. One of them is quinoa (*Chenopodium quinoa*), which is the focus of this review.

## 3 Quinoa as a viable alternative to traditional cereals

Quinoa has been used as a staple food owing to its high nutritional qualities by ancient Andean societies for thousands of years but is still classified as a “semi-domesticated” (Lopes-Marques et al., 2020; Venlet et al., 2021). It is native to Chile, Bolivia, and the Peru mountains and is designated as a “Golden Grain” (Angeli et al., 2020; Yadav et al., 2023). The native geographical region of quinoa extends from south-central Chile to Southern Columbia, along with subtropical Bolivia and north-western Argentina (Costa Tártara et al., 2012). Peru is the main producer of quinoa, followed by Ecuador and Bolivia (Alandia et al., 2020), while France, the USA, and Canada are the leading importers of quinoa. Currently, quinoa is cultivated over 188,000 ha, producing ~175,000 tons per annum (FAOSTAT, 2022; Pathan et al., 2023). In 2020, the global quinoa market was valued at approximately 72 billion USD, and this market is predicted to exceed 149 billion USD by 2026 (Shahbandeh, 2021). Owing to rising demand and its potential to enhance global food security, quinoa farming has recently expanded outside its native Andean region. Quinoa has exceptional nutritional (low glycaemic index; gluten-free; possesses all necessary carbohydrates, lipids, amino acids, vitamins, minerals, protein, and dietary fibres) and has been named by WHO as the “most nutritionally balanced crop on planet” (Angeli et al., 2020). As a result, quinoa cultivation has shifted from just six 50 years ago to over 120 countries now (Alandia et al., 2020). However, global quinoa production represents only a small fraction of the global cereal market (e.g., 175,000 metric tons for quinoa *vs*. 789,000,000 metric tons for wheat in 2022; statista.com).

Most importantly, quinoa is superbly adapted to hostile environmental conditions, including soil salinity, drought, and low fertile soils (Ain et al., 2023) and thus possesses great potential for growth in arid and semi-arid regions of Asia and Africa (Jacobsen et al., 2003). Given the reduced ability of some important cereal crops, including wheat and barley, to survive in arid areas (Houshmand et al., 2005; Aly et al., 2016), quinoa is being grown as a drought- and salt-tolerant alternative in Saudi Arabia, Europe, Morocco, Egypt, and the United States of Emirates (Taaime et al., 2023). Being classified as a halophyte, quinoa in fact *benefit* from the presence of significant amounts of salt in the rhizosphere, showing the highest yield at salinities of 8 to 10 dS/m (Shabala, 2013; **Figure 1C**). Moreover, quinoa yield remains relatively stable across a broad range of salinities (up to 20 dS/m; **Figure 1B**), with some varieties being able to produce significant quantities of grain, even at seawater salt concentrations (Adolf et al., 2013). When benchmarked under future climate scenarios, quinoa yield in 2050 will exceed that of wheat by nearly 3-fold (**Figure 1D**) when grown under irrigated conditions. Under non-irrigated conditions, quinoa *already* outperforms traditional cereals such as wheat in many parts of the world. For example, the 5-year average wheat yield in Australia in 2019–2024 was 2.4 t/ha, (https://ipad.fas.usda.gov/countrysummary/Default.aspx?id=AS&crop=Wheat), with a lowest value of 1.5 t/ha in the driest 2019/2020. The quinoa yield was comparable to or even higher than these values (**Figure 1C**). For example, some quinoa lines achieved the seed yield of 3-3.1 t/ha under rainfed conditions in Western Australia (Dhammu et al., 2019); this is much more than average wheat yield of 1.7 t/ha for the state (Anderson et al., 2016).

## 4 Biological and genetic features of quinoa

Quinoa is primarily an autogamous (self-pollinated) plant with variable rates of natural hybridization (10–17%), which depends on the correlation between flowering and the availability of pollen vectors (Spehar and Santos, 2005; Murphy et al., 2018). It is gynomonoecious (i.e., flowers and female parts are present on the same individual) and possesses numerous small flowers of usually three fundamental types, typically 3–4 mm in size: achlamydeous females, hermaphrodites, and chlamydeous females. These flowers form a panicle-type inflorescence that ranges in length from 15 cm to 70 cm. The structure is highly branched with a central axis that gives rise to secondary and tertiary branches. Manual emasculation for the hybridization of quinoa is difficult because of the presence of small flowers (Zurita-Silva et al., 2014). Certain cultivars exhibit partial or complete male sterility in their flowers, which has been a valuable tool in crop breeding and hybrid development (Bhargava et al., 2006; Gomez-Pando et al., 2019).

Cytological studies have indicated that quinoa is an allotetraploid species with a chromosome number of *2n* = 4x = 36 and a basic chromosome count of x = 9. Quinoa species primarily exhibit diploid and tetrasomic chromosomal segregation (El-Harty et al., 2021). The occurrence of both disomic and tetrasomic segregations at a particular locus is uncommon but can be attributed to the mutual exchange of fragments between homologous chromosomes. Based on morphology, quinoa has been categorized as Chenopodium, subsection *C. berlandieri*, North American diploid *C. watsonii A. Nels*, *C. neomexicanum* Standl., South American tetraploid weed *C. hircinum*, and Andean wild diploid *C. philippianum* (Jellen et al., 2010; Bhargava and Ohri, 2016). The origin of quinoa can be traced to the diploid ancestors *C. carnasolum* and *C. pallidicaule*, and the tetraploid species *C. quinoa* var. *melanospermum* and *C. hircinum* (Mujica and Jacobsen, 2006). The organization and genomic distribution of the 45S nucleolus organizer region (NOR) and 5S ribosomal RNA (rRNA) genes in quinoa have been studied using molecular cytogenetic analysis, which supports this theory. The close relationship between the tetraploid *C. berlandieri* and *C. quinoa* has been confirmed by DNA sequence analysis of NOR intergenic spacers (IGS). Similarly, two distinct non-transcribed spacer (NTS) sequence classes were identified by the characterization of a 5S rDNA spacer region, suggesting that they most likely descended from the two subgenomes of the allopolyploid *C. quinoa* (Kolano et al., 2008).

## 5 Nutritional composition

### 5.1 Proteins and amino acids

Quinoa is enriched with proteins and contains 7-15% higher amounts of essential amino acids and proteins as compared to the major cereals for agriculture and world food such as rice, wheat, and corn (Pathan and Siddiqui, 2022); its protein content is also 14 to 19% higher compared with those species (Bazile, 2023). Moreover, quinoa grains contain albumins and globulins as storage proteins, comprising 44–77% of the total protein content, whereas prolamines make up only 0.5–0.7%, which is significantly lower than in other cereals (Kierulf et al., 2020). The remaining proteins include 1.7% prolamins and 23.16% glutelin, essential for germination (Tavano et al., 2022). Even in small quantities, prolamins are significant for sulphur-rich amino acids. 11S globulin and 2S albumin genes are responsible for the synthesis of these storage proteins (Dakhili et al., 2019). The seeds also contain essential high-quality amino acids, such as lysine, histidine, and methionine, compared to other cereal grains (Bhargava et al., 2009). Based on recommendations for adults, quinoa provides an adequate quantity of essential and non-essential amino acids, such as lysine, tyrosine, cysteine, valine, phenylalanine, and tryptophan. Moreover, sulphur-containing amino acids, such as methionine and cysteine, are particularly present in high amounts at levels comparable to those of soybean and barley, and the histidine protein content of quinoa is higher than that of wheat, soy, and barley proteins (Dakhili et al., 2019). Importantly, while stress conditions such as salinity reduce protein content in wheat and barley by 17% and 21%, respectively, quinoa grains increase protein content by 12% under salinity stress (Aloisi et al., 2016).

Most cereals, including wheat and rice, have a higher gluten content. Gluten intolerance is a serious health issue for many people (Sabença et al., 2021), and high gluten content in food can cause serious health issues for people with non-celiac gluten sensitivity and celiac disease, leading to symptoms such as gastrointestinal distress, skin itchiness, and severe allergic reactions. Quinoa is gluten-free (Satheesh and Fanta, 2018; Villacrés et al., 2022) and ideally suited as a substitute for wheat for protein consumption. Proteins are very complex in structure, and protein digestibility depends on several factors, including the amino acid profile, protein folding, pH, temperature, and ionic strength. One of the problems associated with human-related protein indigestibility is related to structural peculiarities (Becker and Yu, 2013). However, the protein in quinoa grains is highly digestible, with a 91.6% higher absorbance and digestibility (Ruales et al., 2002), which is greater than those other cereals such as corn (36%), rice (56%), and wheat (49%) (Li et al., 2021). In recent years, there has been growing attention in the pharmaceutical industry to the use of quinoa as a source of bioactive peptides (Hernández-Ledesma, 2019). The presence of lunasin, a peptide known for its cancer-preventive properties, in quinoa makes it an attractive plant with pharmaceutical significance (Guo et al., 2023). Furthermore, quinoa contains bioactive compounds that effectively neutralize the strong oxygen radical 2,2′-azino-bis (3-ethyl-benzthiazoline-6-sulfonic acid) and cations (ABTS+) to minimize oxidative damage at the cellular level (Hao et al., 2020). Certain inhibitory effects by quinoa derived bioactive molecules are reported on the production of inflammatory markers like tumour necrosis factor, interleukin-6 (IL-6), and nitric oxide production in lipopolysaccharide (LPS)-stimulated RAW264.7 macrophages (Hao et al., 2020). The RGQVIYVL peptide inhibits the activity of angiotensin-I-converting enzyme and effectively regulates blood pressure. Similarly, other peptides, QFLLAGR and ASPKPSSA, in quinoa also showed iron chelating and free radical scavenging activities, further supporting cardiovascular health.

### 5.2 Carbohydrates

Carbohydrates form the major portion of seed dry weight matter (67-74%) and comprise starch (55-65%), soluble fibres (1.3-6.1%), and dietary fibres (1.1-16.3%) (Filho et al., 2017; Shah and Khan, 2022). Quinoa contains ~3% sugar (mostly D-ribose and D-galactose) as well as minor quantities of fructose and glucose (Mohamed Ahmed et al., 2021). Amylose and amylopectin are primarily responsible for starch synthesis. In quinoa, amylose, which constitutes approximately 11-12% of the starch, is synthesized primarily by Granule-Bound Starch Synthase I (GBSSI), encoded by the GBSSI gene. Amylopectin makes up most of the starch produced through the action of several enzymes, including Starch Branching Enzymes (e.g., SBEI and SBEII), starch synthase (e.g., SSII, SSIII), and starch branching enzymes (e.g., ISA1, ISA2). These enzymes are encoded by their respective genes and collectively contribute to the unique starch properties of quinoa (Srichuwong et al., 2005; Wang et al., 2023). According to Tukomane and Varavinit (2008), quinoa contains 77.5% amylopectin starch content with an average of 317 branching and polymerization degrees of 6700 glucose units per fraction, which is comparable to certain rice cultivars. Compared with starches from other grains, quinoa amylopectin contains a significant number of short chains, ranging from 8 to 12 units, and a smaller number of longer chains, ranging from 13 to 20 units (Satheesh and Fanta, 2018). The presence of both short and long chains in quinoa amylopectin affects its functional properties including digestibility and texture. Amylopectin, with a significant number of short chains, improves the digestibility of starch, which might affect blood sugar levels more quickly, whereas starch with longer chains has low digestibility and glycaemic index (Srichuwong et al., 2017). Moreover, the unique composition of quinoa starch, with shorter and longer amylopectin chains, influences its gelatinization activity. The standard gelatinization temperature for starch usually ranges from 60°C to 75°C (Ai and Jane, 2017). The gelatinization temperature influences the cooking methods, texture, and nutritional properties of grains in various food and industrial applications. The gelatinization temperature range of quinoa starch was observed between 62.6°C and 67°C (Li and Zhu, 2017), which is lower than the gelatinization temperature of rice starch 78°C (Waters et al., 2006).

### 5.3 Fatty acids

The human body requires essential fatty acids from the diet because they are unable to synthesize all the fatty acids. In this context, quinoa is considered to have high quality and quantity of lipid content in its seed oil. Lipid bodies are storage components found in embryo and endosperm cells (Varma and Jain, 2021). The oil content of quinoa varies from 1.8%-9.5% which contains important fatty acids such as oleic acids, alpha-linolenic acid, and linolenic acid, as well as high antioxidant levels α- and γ-tocopherol (Satheesh and Fanta, 2018). Four isomers of tocopherols with different antioxidant properties are present. The oil obtained from quinoa seeds contained a slightly higher concentration of γ-tocopherol than corn germ oil, which contained 251 ppm of α-tocopherol and 558 ppm of γ-tocopherol. Thus, quinoa has a long life because of the antioxidant properties of γ-tocopherol and its high oil content (Maldonado-Alvarado et al., 2023). Moreover, quinoa contains lecithin (1.8%), unsaponifiable matter (5.2%), and sterols (1.5%) and has a refractive index of 1.4637 at 25°C, an iodine value (Wijs) of 129, an acid number of 0.5 (Filho et al., 2017). Furthermore, quinoa oil contains 85% unsaturated fatty acids and 15% saturated fatty acids (Ryan et al., 2007; Satheesh and Fanta, 2018). The stearoyl-ACP desaturase (SAD) gene regulates the overall unsaturated-to-saturated fatty acid ratio in quinoa by converting saturated stearic acid to unsaturated oleic acid (Tupec et al., 2022). Overall, the high proportion of unsaturated fats, particularly polyunsaturated fats, makes quinoa oil a better choice for supporting both health benefits and stability. Triglycerides 85-95% are the most abundant and essential fatty acids in quinoa oil; the rest are made of phospholipids (1-3%), sterols (1-2%), tocopherols (0.5-1%), and free fatty acids (0.1-0.5%) (Pellegrini et al., 2018).

### 5.4 Minerals and vitamins

Quinoa grains contain more Mg, Fe, Ca, Zn, K, Fe, and copper (Cu) than typical cereals, making them a rich source of minerals. K, Ca, Mg, and phosphorus (P) are required in a balanced human diet at levels of 454, 87.4, 190, and 956 mg/100 g, respectively (Brevik et al., 2020). In quinoa, the Ca: P and Ca: Mg ratios are 1:6 and 1:3, respectively, which are greater than the ideal ratios (Tan, 2020). Quinoa has approximately twice the K content relative to wheat, whereas it is four and eight times higher than that of corn and rice, respectively. Similarly, Fe performs various important functions, such as transporting oxygen in red blood cells, and remains the most important micronutrient in the human body; hence, quinoa has three times more Fe content than wheat and five times the content of rice (Vega-Gálvez et al., 2010). Zinc is important for health and participates in various chemical reactions as a cofactor in the body. The Zn content of quinoa is twice and four times higher than that of maize and wheat, respectively, whereas rice lacks this mineral (Filho et al., 2017). Only the Mn concentration was higher in wheat than in quinoa, with rice containing half and only a fifth in maize. Manganese is important for the growth, development, and metabolism of the body (Xiao et al., 2020; Ijaz et al., 2021). However, mineral concentrations appear to change significantly owing to different soil conditions, fertilization treatments, and climate (Gojon et al., 2023).

Furthermore, quinoa is rich in vitamin B6 (0.20 mg/g), vitamin C (1.4 mg/g), folic acid (78.1 mg/g), and pantothenic acid (0.61 g/mg). Quinoa contains vitamin E, vitamins B1 and B2, and α-carotene, which are not present in pseudo-cereal crops. In addition, other vitamins, such as niacin, γ, β-carotene, tocotrienols, and tocopherols, are also found in quinoa seeds. Compared with other pseudo-cereals, quinoa has a high concentration of total folate, riboflavin, vitamin B6, and niacin (Navruz-Varli and Sanlier, 2016; Shah and Khan, 2022). Owing to its rich nutritional profile, integrating quinoa into regular diets can significantly enhance nutrient intake and promote overall health.

## 6 Phytochemicals in quinoa

### 6.1 Betalains

Betalain, a water-soluble phytochemical present in quinoa, acts as a natural antioxidant and contributes to cancer (Samtiya et al., 2021). The quinoa vegetative portion and seeds are colored yellow, black, and red owing to betalain. Red-orange and violet-red betaxanthins that synthesize betalain pigments consist of nitrogen-aromatic indole compounds derived from tyrosine (Hussain et al., 2021). Quinoa varieties with purple or red seed colour typically contain 0.15 and 6.10 mg/100 g betalain (sum of both betacyanins and betaxanthins), which includes both betaxanthins and betacyanins. However, yellow seeds have little to no betalain content. The lack of these pigments results in lighter seed colour (Escribano et al., 2017). The variation in betalain content among quinoa varieties is likely due to genetic differences. Quinoa seeds contain the highest concentrations of isobetanin and betanin, both of which have similar health-promoting properties, such as antibacterial, anti-inflammatory, and antioxidant activities. However, betalain showed greater antioxidant activity than polyphenols. Betalain is the main component of functional foods because of its antibacterial, anticancer, and antilipidemic properties (Calva-Estrada et al., 2022). Recent studies have explored microencapsulation to stabilize betalain and related substances (Aguilar-Tuesta et al., 2018). High betacyanin and low saponin microencapsulation levels exhibit numerous health-promoting attributes, including food colour. The European Union and the United States Food and Drug Administration (US FDA) approved betalain as a natural food color with E-number (E-162) for its utilization in soups, sauces, dairy products, and medicines (Naseer et al., 2019; Hussain et al., 2021). The combination of betalain and saponin is beneficial for both food and pharmaceutical applications (Esatbeyoglu et al., 2015).

### 6.2 Phytoecdysteroids

Phytoecdysteroids are secondary metabolites that protect plants from insect pests, whereas nematodes are cyclopentanoperhydrophenanthrene-ringed polyhydroxylated chemicals. Its structural makeup varies significantly depending on the number of carbon atoms present in the structure. They are classified as C27- and C28-phytoecdysteroids and are mainly located in the main region of the grain as polar/non-polar and free-conjugated compounds (Dinan et al., 2021). Quinoa is the only pseudo-cereal with a considerable concentration of phytoecdysteroids, ranging from 138 μg/g to 570 μg/g (Hussain et al., 2021). The quinoa plant contains approximately 36 different types of phytoecdysteroids, with the highest concentration found in C27 phytoecdysteroids, which offers numerous health benefits (Graf et al., 2016). For instance, phytoecdysteroids exhibit antioxidant potential because of their free radical scavenging activity and metal ion chelating ability, making them useful for preventing skin aging (Lin et al., 2019; Sidorova et al., 2022). Unlike synthetic anabolic steroids, which can cause significant health risks, phytoecdysteroids provide a non-toxic alternative for athletes and bodybuilders that naturally enhances protein synthesis and supports muscle growth (Báthori et al., 2008). These bioactive substances also play a significant role in promoting the growth of skeletal muscles, which is crucial for enhancing physical performance (Báthori et al., 2008). Similarly, numerous *in vivo* studies have demonstrated the effectiveness of quinoa phytoecdysteroids in combating obesity. A previous study revealed that incorporating quinoa extract into a high-fat diet helps in obesity management (Cao et al., 2020). Dietary administration of quinoa resulted in decreased fat mass, mainly through increased faecal defecation of lipids and carbohydrate oxidation. Furthermore, quinoa phytoecdysteroids may help prevent diabetes by reducing oxidative degradation and improving blood glucose transport in the blood (Zang et al., 2024).

### 6.3 Phenolic compounds

Natural organic molecules, also known as phenols, consist of aromatic rings linked to one or more hydroxyl groups. They can be divided into two subgroups, simple and complex phenols, depending on the presence of benzene rings (Abbas et al., 2017). The cell walls of quinoa leaves contain phenolic acids in both the free and chemically bound forms (Hernández-Ledesma, 2019). Numerous types of phenolic acids, including hydroxybenzoic acid and hydroxycinnamic acid, are found in quinoa seeds and leaves, and have significant health-promoting properties, such as anticarcinogenic, antioxidative, antihypertensive, and antidiabetic properties. Most polyphenols in quinoa are flavanol-type flavonoids, primarily quercetin, kaempferol, and their derivatives (Balakrishnan and Schneider, 2020). Rutin, morin, neohesperidin, vitexin, and other flavonoids have been linked with quinoa (Paśko et al., 2008). Numerous studies have shown that the total phenolic content of quinoa increases considerably after germination. Germination activates enzymes in quinoa seeds that break down stored compounds, resulting in the release and synthesis of phenolic compounds (Darwish et al., 2021; Liu et al., 2020b; Bhinder et al., 2021). The total phenolic content of quinoa is affected by color, genotype, and growing conditions (Han et al., 2019). Flavanol glycosides (Gómez-Caravaca et al., 2011). Quinoa contains 12 distinct types of flavanol glycosides, mainly derivatives of kaempferol and quercetin, with an average individual concentration of 839 μg/g on a dry-weight basis (Li et al., 2021).

### 6.4 Saponins

In the Plant Kingdom, saponins are present in at least 400 plant species from 40 different families. The term “saponin” is derived from the Latin word “sapo,” meaning soap, because of its surfactant properties that allow it to produce persistent, soap-like foam when agitated in an aqueous solution. Saponins decrease vitamin absorption and form complexes with sterols that are structurally similar to fat-soluble vitamins, which further interferes with vitamin absorption and sterol activity (El Hazzam et al., 2020; Otterbach et al., 2021). The bitter taste of saponins makes them poisonous in large quantities. The presence of saponins in quinoa is commonly believed to serve as a defence mechanism against natural enemies owing to their bitter and toxic properties (Dong et al., 2020). For example, a crude saponin extract of *C. quinoa* has antifungal properties (Pillimué et al., 2024). Moreover, quinoa saponins significantly improve the germination rate of rice seeds and show a biocidal effect against the rice seed-eating snail, *Pomacea canaliculata* (Lin et al., 2019; Zaynab et al., 2021). Recent genetic studies have identified the key genes involved in the regulation of saponin metabolism in quinoa. Similarly, expression analysis in quinoa has revealed that *TSARL1* is mainly expressed in immature seeds and flowers (Jarvis et al., 2017; Trinh et al., 2024), whereas *TSARL2* is expressed only in root tissues (Trinh et al., 2024). The TSAR binding motif has been detected upstream of various genes in the saponin biosynthetic pathway in quinoa. A comparison of *TSARL1* transcripts between bitter and sweet quinoa accessions identified an alternative splicing event in sweet accessions that resulted from a single nucleotide polymorphism (SNP) affecting the intron/exon splicing boundary. In sweet quinoa varieties, further analysis of *TSARL1* showed various independent gene mutations that co-segregated with the sweet phenotype, suggesting that this gene controls saponin levels in seeds (Jarvis et al., 2017; Otterbach et al., 2021; Trinh et al., 2024).

Numerous biological, chemical, and physiological properties of saponins are known, including hemolytic, anti-inflammatory, antibacterial, antiviral, and cytotoxic effects (Tan et al., 2022). Like, four fractions of quinoa saponins Q30, Q50, Q70, and Q90 extracted from quinoa have shown anti-inflammatory effects. The saponin fractions inhibited the production of inflammatory cytokines such as interleukin-6 and tumour necrosis factor in lipopolysaccharide-induced RAW264.7 cells (Wijesekara et al., 2024). Another study showed that quinoa saponins enhance antibody responses (IgA and IgG) by increasing mucosal permeability for greater antigen absorption (Yao et al., 2015). Moreover, they affect the absorption of specific minerals and vitamins as well as the growth of eating organisms (San Martín et al., 2008). Currently, 40 saponins have been reported, and the four major sapogenins of quinoa are phytolaccagenic acid, hederagenin, 30-O-methyl-espergulagenate, and oleanolic acid (Otterbach et al., 2021). In quinoa, saponin concentrations vary from 0.1% to 5.0%. Quinoa can be bitter or sweet, depending on the quinoa variety and saponin concentration. They can be used in the production of beer, detergents, cosmetics, fire extinguishers, and immunologic adjuvants in vaccines (Tan et al., 2022).

## 7 Factors affecting nutritional composition of quinoa

In recent years, farmers have been encouraged to cultivate quinoa instead of traditional crops because of their nutritional benefits and increasing demand in the global market. The crop requires appropriate agronomic practices such as optimal crop geometry, water management, high-yielding cultivars, correct nutrition, and proper harvesting to ensure enhanced nutritional components and gain yield. Agronomic approaches employed in crop production systems can regulate plant development (i.e., shelf life, colour, texture, and appearance) by improving grain quality attributes and altering the transcriptome.

### 7.1 Sowing date

Sowing is a crucial agrotechnology, as seedling emergence affects the plant population, grain quality, and yield. In mid-October in India, November-December in Morocco, and mid-November in Bhutan are the preferred sowing times for quinoa cultivars with high yield (Ramesh et al., 2019; Dorji et al., 2020; Taaime et al., 2022). These conditions decreased the risk of heat stress and ensured better grain filling to achieve higher yields. Rathore et al. (2019) observed that the proximal composition of quinoa seeds is affected by sowing time. Higher protein and fat contents were observed in quinoa seeds sown in January, likely because of thermal conditions during grain filling. Late sowing increases air temperature and reduces rainfall, resulting in less nitrogen leaching from the soil and a gradual increase in protein content (Zulkadir and Idikut, 2021). The protein content in quinoa varies from 13.5% to 17.7% based on the different sowing dates (Temel and Keskin, 2019). Moreover, variations in sowing time and rising air temperatures resulted in an increase in Mg and P levels in late sowing; however, this increase declined in late sowing (Zou et al., 2021). This decline was attributed to the grain-filling and flowering periods of quinoa, a short-day plant, coinciding with the maximum day length and high temperature (Zulkadir, 2021). Similarly, variations in P and Mg contents have been reported in quinoa varieties (Kaya and Aydemir, 2020; Gómez et al., 2021). When cultivated at the Research and Didactic Station in Psary, early August-sown quinoa accumulated 39.4% less N-NO_3_ and 44.4% more P. A gradual delay in harvest results in an increase in Mg and Ca levels and a reduction in P content among different quinoa varieties (Liu et al., 2021; Adamczewska-Sowińska et al., 2021).

### 7.2 Temperature

Temperature is the primary abiotic factor influencing quinoa germination, development, quality, and production. The ideal temperature for quinoa germination is approximately 8-10 °C (Ayala et al., 2020); however, it grows well in later stages at temperatures ranging from 15 °C to 20 °C and can even tolerate extreme temperatures − 8 °C or 38 °C (Ain et al., 2023). The fatty acid content of quinoa is directly affected by temperature (Matías et al., 2022). Linolenic acid is a major fatty acid found in higher contents at the seed-filling stage in quinoa due to elevated temperatures. This temperature-dependent response is similar to the response observed by Curti et al. (2020), who demonstrated that most of the quinoa cultivars produced less polyunsaturated fatty acids (PUFA), including oleic acid, under high temperature (except for linolenic acid). The increase in the concentration of unsaturated fatty acids in quinoa seeds at high temperatures during the seed-filling stage might be due to the temperature-sensitive nature of fatty acid biosynthesis. Elevated temperatures also accelerate the enzyme activity involved in the desaturation process, particularly the conversion of saturated fatty acids to polyunsaturated fatty acids such as linoleic acid. The increase in enzymatic activity changes the equilibrium towards the production of linoleic acid (Song and Tang, 2023). Matías et al. (2022) also reported changes in the fatty acid composition with significantly lower monounsaturated fatty acids with changing temperature, whereas there was no change in the overall amount of saturated fatty acids. The various impacts of temperature on the concentrations of seed elements in quinoa were also revealed such as, plants grown at a shoot temperature of 22 °C and root temperature of 30 °C exhibited lower concentrations of elements such as Cd, B, As, Al, Rb, Sr, Cu, and Ni (Tovar et al., 2020).

### 7.3 Fertilizers and water management

Quinoa is known for its low nutrient requirements and ability to grow in impoverished soils (Jacobsen et al., 2003; Adolf et al., 2013). However, N fertilization can improve the quinoa’s ability to grow under drought conditions (Bascuñán-Godoy et al., 2018; Zamani et al., 2023) and further boost the nutritive value of quinoa by improving protein content in grains and enhancing P, K, and N concentrations in quinoa (Ibrahim et al., 2020). Gomaa (2013) and Wang et al. (2020) reported that appropriate N supply was the dominant factor for protein accumulation in quinoa seeds. N fertilization significantly affects the chemical traits of quinoa, including the contents of N, P, K, total protein, starch, fat, and ash (Wali et al., 2022; Van Minh et al., 2022). Vermicompost serves as an organic nutrient source for quinoa cultivation. Applying 5 tons/ha of vermicompost results in an increase in quinoa grain output along with higher crude fiber, fat, and carbohydrate (Rathore and Kumar, 2021).

The water requirement of quinoa is low, and it can tolerate dry spells, yet irrigation has a significant impact on quality and productivity (Angeli et al., 2020). Different irrigation treatments influenced the concentrations of several seed minerals in quinoa, including Cu, Ca, Mg, P, Fe, and Zn. The concentrations of Mg, Fe, and P were enhanced under irrigated conditions, whereas Zn, Ca, and Cu concentrations increased under drought conditions (Walters et al., 2016). One reason for the difference in mineral concentrations between water-deficient and irrigated plants might be the variations in seed size and plants. Irrigation at crucial stages, such as germination, seed set, and initial flowering, ensures high mineral concentrations and maximum grain yield. In the last two decades, metabolomics has emerged as a robust molecular profiling technology, and its results can be integrated with data from other technologies (Riekeberg and Powers, 2017; Tong and Nikoloski, 2021). These developments have enabled the identification and annotation of previously unknown metabolites as well as the documentation of underlying biochemical reactions and associated enzymes (Sharma et al., 2021).

## 8 Unanswered questions and a way forward

As shown in this study (**Figure 1**), the use of traditional cereal crops such as wheat or rice will be severely hampered by future climate scenarios, making their production economically unfeasible and unsustainable. Quinoa (*Chenopodium quinoa*) has a great potential to occupy this niche. However, before this can happen, several issues need to be resolved. These can be roughly divided into two categories: biological and social.

### 8.1 Biological aspects

From an agronomical perspective, quinoa can tolerate both salinity and drought stress and is ideally suited for cool-climate production systems. However, originating from high-altitude regions in South America, it is less suitable for production at high temperatures (unless irrigation is used). This trait needs improvement, along with its photoperiodic sensitivity (most quinoa genotypes have a requirement for a short day), tolerance to downy mildew, and several yield-related components, such as seed size and shattering and pre-harvest sprouting (Lopes-Marques et al., 2020). High quantities of saponins are also considered a major hurdle, as they imply an extra step in food processing. Improving these traits using genetic means will accelerate the broader use of quinoa in agricultural production systems. With over 16,000 quinoa accessions existing and given the availability of several high-quality genome drafts (Jarvis et al., 2017; Zou et al., 2017), such genetic improvement should be rather straightforward and are only hindered by the lack of dedicated funding. The task is not trivial but still much easier than regaining abiotic stress tolerance in traditional cereal crops (see arguments in Palmgren and Shabala, 2024).

In broad terms, the above tasks can be divided into short-term (1–5 years) and long-term (over 5 years). The former should focus on applying existing genomic tools to address immediate challenges in quinoa production. Marker-assisted selection (MAS) can be employed to rapidly introgressed known disease-resistance genes, such as those conferring resistance to downy mildew or fungal pathogens, into elite quinoa lines. Similarly, high-throughput phenotyping and genomic screening can identify accessions with desirable abiotic stress tolerance traits such as salt tolerance mediated by *NHX1* transporters or drought resilience linked to osmoprotectants biosynthesis enabling faster development of climate-resilient varieties (Lozano‐Isla et al., 2025). These efforts can be accelerated by leveraging publicly available quinoa genome assemblies (e.g., *Chenopodium quinoa* v1.0) and trait-associated SNP markers from recent genome-wide association studies (GWAS) (Maldonado-Taipe et al., 2022; Rahman et al., 2024). For the medium- and long-term objectives, genomic selection (GS) and gene editing hold significant promise. GS models trained on large-scale quinoa germplasm datasets can predict breeding values for complex traits like yield stability, protein content, and saponin levels, reducing reliance on time-consuming phenotypic selection (Kumar et al., 2021). Additionally, CRISPR-Cas9 could be used to precisely edit genes involved in anti-nutritional factors, such as the *CYP72A* family responsible for bitter saponin production, facilitating the development of “sweet” quinoa varieties without lengthy backcrossing (Liu et al., 2019). Hybrid breeding programs could also benefit from genomic insights by identifying heterotic groups and optimizing cross-combinations using SNP-based genetic distance analyses. Quinoa improvement may also shift toward transformative genetic engineering and synthetic biology approaches. For example, metabolic engineering of lysine biosynthesis pathways (*AK1*, *DHDPS*) could improve quinoa’s protein quality, making it a more complete dietary staple (Kiekens et al., 2022). Exploring the genetic diversity of wild relatives (e.g., *C. berlandieri*) through *de novo* domestication could also unlock novel alleles for extreme heat tolerance or pest resistance. These efforts will require advanced genomic resources, such as a fully annotated pan-genome encompassing global quinoa diversity, as well as interdisciplinary collaboration between breeders, bioinformaticians, and biotechnologists.

In parallel to promoting quinoa as a potential substitute for traditional cereals, some of its key traits conferring superior abiotic stress tolerance may also be potentially introduced in wheat and rice (Chen et al., 2022; Shabala and Palmgren, 2020). It was argued that the traditional focus of breeders on targeting SOS1 and HKT1 genes to improve salinity stress tolerance in these species is counterproductive (Shabala et al., 2025) and called for a need for a major shift in a breeding paradigm to incorporate some halophytic traits that were present in wild relatives but were lost in modern crops during domestication (Rawat et al., 2022). Amongst the latter, efficient vacuolar Na^+^ sequestration, ROS desensitization, succulence, and a possibility for salt deposition in trichomes have been named as most promising novel traits. In this context, quinoa represents an ideal model species, ticking on all “boxes” such as possessing a superior ability to prevent back-leak of toxic Na^+^ from vacuole into cytosol by efficient control of slow (SV) and fast (FV) vacuolar ion channels (Bonales-Alatorre et al., 2013a, b); reduced sensitivity of K^+^ transporters to ROS (Tanveer et al., 2024); and ability of deposit substantial amount of salt into epidermal bladder cells (Kiani-Pouya et al., 2017; Bohm et al., 2018). By using modern breeding technologies, these features may be incorporated into cereal elite germplasm, to regain abiotic stress tolerance.

### 8.2 Social aspects

The global expansion of quinoa over the past few decades highlights its strong potential for economic scalability, which is a critical factor supporting its adoption by farmers. The number of countries importing quinoa is increasing, with new producers emerging and cultivation expanding outside the Andean regions. This growing geographical distribution reflects quinoa rising importance in the international market. The increase in demand for gluten-free, high-protein foods across North America, Europe, and Asia has created new opportunities for farmers to adopt quinoa as a profitable crop. Moreover, the development of value-added products such as quinoa flour, starch, milk, and processed snacks has further enhanced its commercial application.

Importantly, quinoa is primarily cultivated by smallholder and family farms in both traditional and newly adopting regions, emphasizing its suitability for scalable, low-input farming systems. Countries such as China, Canada, and France have increase significant growth in quinoa production, scaling up from fewer than 100 farmers in 2012 to hundreds by 2018, with China alone reaching 17,000 hectares by 2019 (Xiu-Shi et al., 2019). In many new production areas, quinoa is introduced to diversify cropping systems or replace less resilient crops an approach driven to adapt agriculture due to increasing impacts of climate change. However, to sustain this growth and ensure long-term farmer engagement, it is essential to establish stable market channels and secure favourable pricing to enhance farmer incomes and encourage continued cultivation.

The social aspects of this issue should not be underestimated. Rice is a traditional food for more than half of the world’s population, especially in Asia and Sub-Saharan Africa, and replacing it with quinoa may clash with many traditional values and habits. The same may be true for wheat as well. Thus, the acceptance of quinoa as a major staple food requires time. Recent surveys conducted in various regions have provided a clear link between cultural background and consumer acceptance of quinoa. The survey of 381 peoples in Lima revealed that individuals with stronger ethnic identities were less willing to consume quinoa, suggesting that cultural perceptions and traditional food preferences play a crucial role in dietary choices (Higuchi et al., 2022). Similarly, Wang et al. (2024) conduct survey analysis of 1078 individuals in 16 different administrative districts of Shanghai and demonstrated that only 38.22% of individuals have purchased quinoa products. The trust in nutrition and personal norms significantly influenced the willingness to purchase quinoa products. In Europe, consumer acceptance of quinoa is mostly influenced by health perceptions, dietary habits, and product availability. A cross-sectional survey conducted in Italy analyzed the nutritional quality of quinoa food products available in the market and found that consumers are attracted to quinoa due to its nutritional benefits, gluten-free nature, and ethical rights associated with its production (Melini et al., 2023). These findings underscore the importance of cultural factors in consuming quinoa in market, as acceptance is not only based on nutritional value but also on cultural perceptions and compatibility.

Importantly, this cultural shift should come from the grassroots rather than being imposed from the top. In lay terms, farmers need to realize that the production of traditional cereal crops cannot be considered a long-term strategy, due to economic reasons, and make this paradigm shift on their own. Appropriate government incentives may accelerate this process.
